# Extreme immunotherapy: emergency immunology to defeat pandemics

**DOI:** 10.1186/s10020-021-00366-4

**Published:** 2021-09-16

**Authors:** Douglas F. Nixon, Daniela Marín-Hernández, Nathaniel Hupert

**Affiliations:** 1 Division of Infectious Diseases, Department of Medicine, Weill Cornell Medicine, Belfer Research Building, Room 530, 413 E. 69th Street, New York, NY 10065 USA; 2 Department of Population Health Sciences, Weill Cornell Medicine, 402 E. 67th Street, New York, NY 10065 USA; 3 Cornell Institute for Disease and Disaster Preparedness, Weill Cornell Medicine, 402 E. 67th Street, New York, NY 10065 USA

**Keywords:** COVID-19, SARS-CoV-2, Pandemic, Immunotherapy, Emergency

## Abstract

The ongoing global COVID-19 pandemic has thrown into sharp relief the gap between modern biology’s ability to investigate and respond to a novel pathogen and modern medicine’s ability to marshal effective front-line interventions to limit its immediate health impact. While we have witnessed the rapid development of innovative vaccines against SARS-CoV-2 using novel molecular platforms, these have yet to alter the pandemic’s long-term trajectory in all but a handful of high-income countries. Health workers at the clinical front lines have little more in their clinical armamentarium than was available a century ago—chiefly oxygen and steroids—and yet advances in modern immunology and immunotherapeutics suggest an underuse of extant and effective, if unorthodox, therapies, which we now call “Extreme Immunotherapies for Pandemics (EIPs).”

## Background

This perspective takes the position that there will be future pandemics, or epidemics with potential to turn into pandemics. What might cause these pandemics in the future is based upon the history of past and present epidemics, with strategic consideration of the future, plus the unknown “X’ Factor. The WHO provides a list of current potential pathogens, under its R & D blueprint list of prioritizing diseases for research and development in emergency contexts, and these include: COVID-19; Crimean-Congo haemorrhagic fever; Ebola virus disease and Marburg virus disease; Lassa fever; Middle East respiratory syndrome coronavirus (MERS-CoV) and Severe Acute Respiratory Syndrome (SARS); Nipah and henipaviral diseases; Rift Valley fever; Zika and ‘Disease X’ (WHO 2021). Disease X represents the knowledge that a serious international epidemic could be caused by a pathogen currently unknown to cause human disease (WHO 2021).

We have learnt much from the COVID-19 pandemic, including what was successful in terms of physical interventions, non-pharmaceutical interventions (Ragonnet-Cronin et al. 2021; Spinelli 2021), and the incredible success of vaccination (Haas 2021), but therapeutic interventions have largely been ineffective, and no blockbuster antiviral drug has been developed or used (WHO 2021). As understanding of the pathology of the disease developed, a broad breakdown into an anti-viral followed by an anti-inflammatory phase has driven drug studies (Fergie and Srivastava 2021), yet current clinical trial design seems inadequate for rapid decision making (Pearson 2021). We still do not have the studies to tell whether common non-specific agents have some general effect in COVID-19, while drugs that were widely used such as hydroxychloroquine or ivermectin only now are known to not have a beneficial effect (WHO 2021; Bartoszko 2021). Specific anti-viral agents such as remdesivir appear to have only a minor impact on COVID-19 disease course but not on overall mortality (Beigel et al. 2020). The use of the steroid dexamethasone has been shown to assist in late stage illness thought to be due suppression of exuberant inflammatory responses (Group et al. 2021), yet therapeutics for individual disease presentations is still one-size-fits-all, and there has been few front-line advances in “precision clinicopathological correlation” of treatment options.

**Fig. 1:**
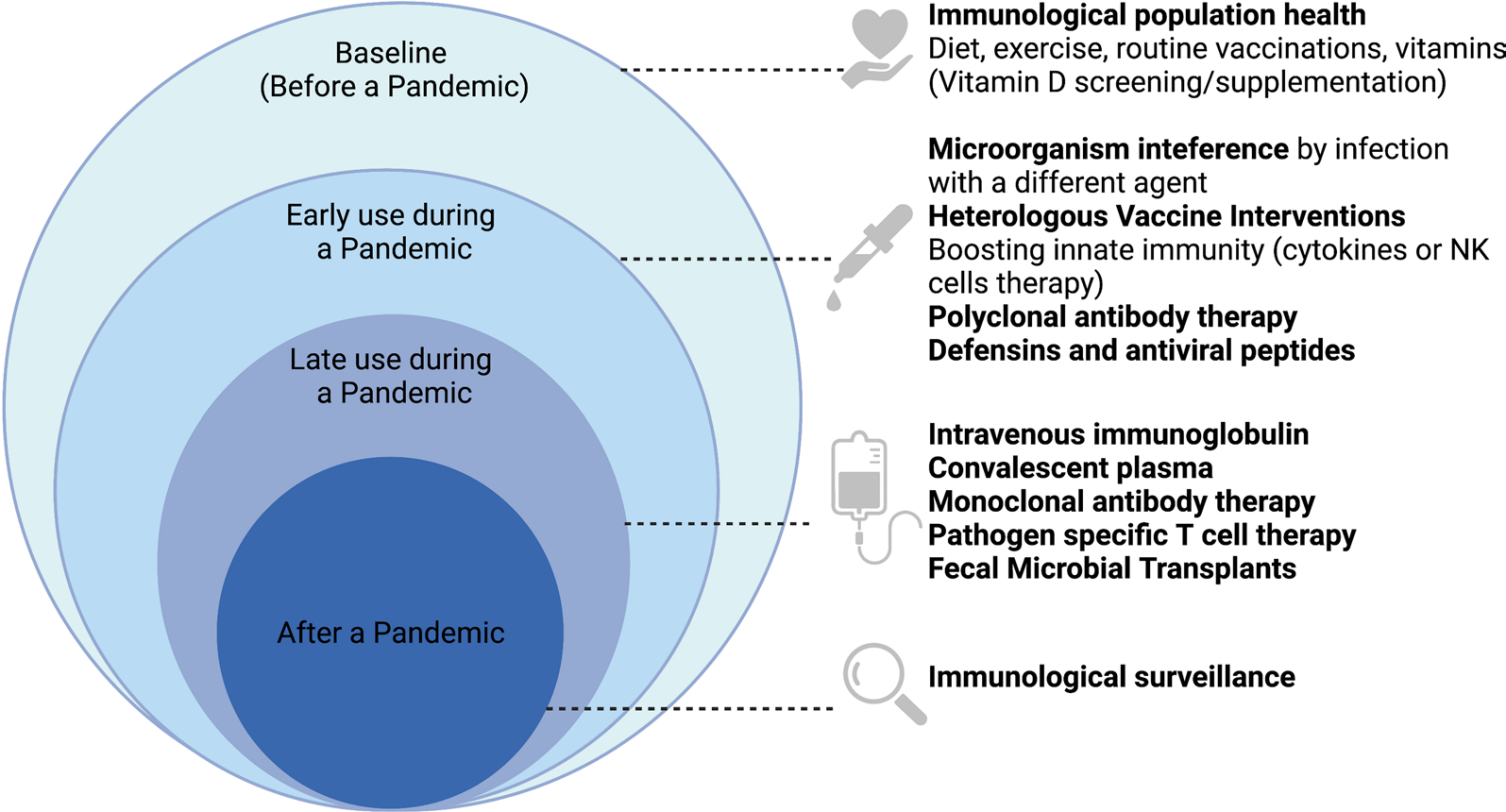
Extreme immunotherpies for pandemics timeline

In this setting, we sought to review the availability and potential impact of emergency and extreme immunotherapeutic interventions for COVID-19 and future pandemic pathogens, which we call “Extreme Immunotherapies for Pandemics (EIPs)”. From an immunotherapy perspective, there may be solutions not well discussed ranging from non-controversial to extreme that could be used to potentially combat a new pandemic, either at a population level or an individual level. If a pandemic pathogen were spreading quickly, widely, and without control measures, emergency measures would be needed to help curb morbidity and mortality. This perspective brings our ideas on EIPs for future pandemics, and attempts to highlight how specialized therapies might be adapted for more widespread use (Fig. 1). We acknowledge that some of the ideas in this perspective are controversial and would require further conceptual clarification and explanations on how they would be implemented in the case of a new pandemic, but we want to help start that essential dialogue—before any new pandemic.

### Before pathogen identification

Emergency immunology and immunotherapy comes in to play at the earliest stage of a pandemic discovery: how can the population immunological health be immediately rapidly boosted? How do individual differences in genetics and environmental exposure impact mass implementation? And how can mass or individual immunotherapy be used once a pandemic has started? As we have seen with the evolution of virus variants in the current COVID-19 pandemic, can immunotherapy for variant escape be used as a holding mechanism while variant vaccines are rapidly developed?

There are some general principles which could be considered: immunological health is likely related to overall population health and general strong advice on good diet, increased exercise, and review of routine vaccination status could all lead to an ‘improvement in overall and immunological health’, although there as of yet are no standards for the immunological pulse or *terrain* of a general population (Marin-Hernandez et al. 2021). While the real impact of vitamins is unclear, screening and supplementation of Vitamin D in high risk groups, especially in the elderly, should be considered as a measure to improve general immunological health (Chambers et al. 2021).

Despite intense efforts to mitigate the COVID-19 pandemic, the third or fourth waves of infections and new cases around the world suggest that politics, fatigue, lack of preparedness, lack of resources and infrastructure (including basic hospitals) especially in low income countries, will continue to prolong this pandemic for some time. Yet, there are bystander effects on population level infectious disease health that are still being discovered, like the extremely low prevalence of influenza A infections this year in both the Southern and Northern Hemispheres. Epidemiological studies have shown ecological interactions between infections for some time (Opatowski et al. 2018; Nickbakhsh 2019), and for a future pandemic, one extreme immunological intervention would be to deliberately infect the population with another agent which would either be less pathogenic or occupy a niche to prevent target cell availability, (under the competitive exclusion principle). To be effective, a high percentage of target cells would need to be infected for a long period of time, but by inducing IFNs, a general antiviral effect could be achieved. While this has not been tried on a large scale, attenuated vaccines work on a similar principle, and in vitro studies have identified ‘viral interference’ in target cells (Dianzani 1975). There are important ethical considerations that need to be considered and discussed around giving a less pathogenic agent to help protect against a lethal pathogen. Indeed, vulnerable individuals (i.e., elderly, immunosuppressed) might need to avoid the ‘less’ pathogenic agent which might cause disease in the vulnerable. There would also need to be close monitoring of such an approach, as adaptation of the pathogenic agent to a broader host range would be highly deleterious.

Commonly used vaccines can have nonspecific effects, including heterologous effects against SARS-CoV-2 infection. We, and others, have proposed Heterologous Vaccine Interventions (HVIs) as interventions for reducing viral pandemic-related morbidity and mortality (Marin-Hernandez et al. 2021; Hupert et al. 2021). The BCG vaccine in particular has been associated ecologically with non-specific benefits even long after immunization, and as an agent to “boost” innate trained immunity which could act against a range of potential pandemic pathogens (Moorlag et al. 2019), it seems to be the most valuable, although other vaccines should not be ignored. Perhaps one of the first recommendations when a pandemic is on the horizon is to have the population catch up with routine, age-specific vaccination regimens.

We take the position that in order to help prevent infection or reduce the magnitude of a disease, in almost all cases strengthening the innate component of an individual’s immune system would help. Once infection with a new pathogen has occurred, boosting immunity as soon as possible through agents such as IVIG (see section below), or innate cell therapy potentially could reduce mortality. Natural Killer (NK) cell responses could be boosted by administration of cytokines such as IL-12/IL-18, IL-15, and cytokines have been given to patients with a number of conditions including patients with cancer (Wrangle et al. 2018). Cytokine administration is an exciting area for exploration, but also not without potential side effects (Rosenberg 2014). NK cells could also be boosted through bystander actions such as giving an influenza vaccine which has been shown to non-specifically increase NK cells post vaccination (Long et al. 2008; Ram et al. 2019) (Market et al. 2020). A more complex way to provide NK cells would be through the use of cellular therapy with “off the shelf” NK cells (Ram et al. 2019). As more is learned about “innate NK cell memory” and “trained immunity”, cross reactive NK cells to another related pathogen might be elicitable in the future.

Other innate cells, gamma delta, MAIT and NKT cells could also be targeted through ligand engagement or direct cell therapy with expanded cells. Each of these cell types might have a preferential role depending on the pandemic pathogen (Poccia et al. 2005; Pistoia et al. 2018; Wakao et al. 2017; Manna et al. 2020; Wang, et al. 2020), and immunologists should be a constant part of the rapid pandemic response scientific team (Marin-Hernandez et al. 2021). Interferon therapy could be considered in some viral infections (Calabrese et al. 2020). Apart from antiviral activity, interferons possess antiproliferative properties and can modulate cell differentiation. Depending on the desired outcome, antiviral/antiproliferative activities or immunomodulation, high or low doses, and of different interferon classes, can be used, respectively. In the treatment of herpes zoster, high daily doses of interferon achieving high serum levels for several days has been described (Brzoska et al. 2020).

### Immunological identification of the pandemic pathogen

We think that the identification and isolation of a pandemic pathogen would be reasonably straightforward, with a list of current contenders the most likely to spread. However, there is a chance that a truly new virus or pathogen might emerge, and molecular characterization of the human immune response might be useful to “reverse identify” a family of potential pathogens, through systems immunology identification of a specific immune response (Davis et al. 2017). This could be through BCR or TCR repertoire analyses identifying group pathogen specific receptor usage, or unique and distinct signatures pathogen gene-expression analysis. We consider this a use of emergency systems immunology.

### After pathogen identification

Once a pathogen has been isolated and sequenced, specific antibodies can be generated by molecular means or through animal immunization such as with highly purified F(ab′)2 fragments of horse specific polyclonal immunoglobulins. Equine polyclonal antibodies containing highly purified F(ab′)2 fragments are safe and well tolerated, and easy to manufacture (Zylberman et al. 2020), and could buy time before other antibodies are isolated or created.

Administration of intravenous immunoglobulin (IVIG) could also be rapidly utilized. IVIG is a blood product prepared from the serum of between 1000 and 15,000 donors per batch, offers a broad antimicrobial activity, and has been found to be effective in reducing the number of bacterial infections when given prophylactically to patients with chronic lymphocytic leukemia (Long et al. 2008), or in human enteroviral encephalitis, but the timing and route of IVIG administration would be important. At a later stage of a pandemic a controlled trial of IVIg vs. convalescent plasma vs. control would be a definitive study, but again difficult to do under current clinical trial guidelines.

The use of pooled immunoglobulin against SARS-CoV-2 failed in clinical trials, but combination monoclonal antibody therapy has been adopted with some success at an early enough stage to help reduce hospitalization rates (Kumar et al. 2021), although surveillance for variants and monitoring of effectiveness of monoclonal antibodies (mAbs) needs to be a continuous process (Boskovic et al. 2021). The timing of humoral interventions are probably critical. In COVID-19 the relatively early emergence of neutralizing antibodies (NAbs) (within the first 14 days) is associated with a beneficial effect for future recovery (Lucas 2021). MAb therapy or convalescent plasma work best when the NAbs are present in large quantities and as early as possible, so as to suppress viremia when it matters most (Honjo 2021; Klassen et al. 2021). This is likely to be a generalizable statement for other pathogens causing pandemics. Adoptive pathogen specific T cell therapy could also be used, and although the time it takes to generate virus specific T cells has decreased, this type of therapy may be less useful for acute pathogens (Keller et al. 2020). Small animal models may become more useful in the future for assessment of virus immunotherapies (McCann et al. 2021).

Attenuated varicella virus vaccine (Zostavax) has been given to prevent disease symptoms from Varicella virus infection (Bruxvoort et al. 2019), so for a new pandemic pathogen, attenuation of the pathogen and administration could be trialed. This would require: (1) that the pathogen has been identified, (2) isolated, and (3) passaged in a manner causing mutations that decrease pathogenesis while still inducing protective immune responses. This is unlikely to be achieved in a short time frame. There are, of course, significant potential risks of this approach, but also potential rewards.

### Emergency immunotherapy

Although un-conventional and not approved, there are some therapeutic options which could be considered in dire circumstances:

A. Fecal Microbial Transplants/Treatment with short-chain fatty acids and tryptophan metabolites
  In a recent study, mice that recovered from high-dose radiation to normal life spans were elite survivors who harbored a distinct gut microbiota, and metabolomics showed increased metabolites in elite survivors. When administered to other mice, long-term radioprotection, mitigation of hematopoietic and gastrointestinal syndromes, and a reduction in proinflammatory responses were observed (Guo, et al. 2020). We speculate, analogously, that “elite survivors” of pathogenic infections may have unique microbial or metabolite signatures that could be used to help protect others. Fecal microbial transplants are not without potential serious side effects, and would need to be considered carefully before use, with full transparent ethical considerations.
B. Defensins and antiviral peptides
  Defensins are a major family of host defense peptides expressed predominantly in neutrophils and epithelial cells. Their broad antimicrobial activities and immunomodulatory functions have been extensively studied, and they are considered a core host-protective component against bacterial, viral and fungal infections (Xu and Lu 2020). However, other studies have suggested potential pathogenic effects, including viral enhancement (Xu and Lu 2020), and clinical studies proceed with caution (Dijksteel et al. 2021). However, given the alteration in risk–benefit calculations during the acute phase of pandemic response, we would argue for their consideration within the scope of EIPs.
C. Baboon bone-marrow xenotransplantation
  In 1995, a person living with HIV (PLWH) who had failed to respond to triple-drug antiretroviral therapy was given a Baboon bone-marrow xenotransplant as a ‘last ditch’ attempt to find a new approach to killing HIV infected cells, and replacing target cells with resistant cells (Starzl et al. 1993; Relf 1996; James 1995). Although unlikely to be used in an acute pandemic situation, this highly unconventional, last resort approach paid off for this patient, and it is this type of bold out of the box thinking that we encourage the research and clinical community to consider as last resort approaches when considering EIP candidates.

## Conclusions

### An immunological therapeutic tool box

We find it notable that during the COVID-19 pandemic, there were no professional immunologists sitting on the U.S. Advisory Committee for Immunization Practices (ACIP), one of the most respected bodies in the world advising not only on the use of pandemic vaccines but more generally on the theoretical framework for prioritizing pandemic interventions. We believe that immunologists should have a clearly designated role in pandemic preparedness and response (Marin-Hernandez et al. 2021). As our global response to COVID-19 continues, and certainly before any future pandemic, immunology bodies can champion research into animal models for rapid testing of immunological agents; the immunology of anti-pathogen immune responses in mice, hamsters and ferrets should be a high priority investment area. The immunological surveillance of populations at a systems immunology level should be developed. Immunological solutions to infectious pathogens should be considered as part of a planned pre-pandemic therapeutic development and we suggest the creation of an ‘immunological therapeutic tool box’ (Table 1), which could be rapidly deployed as part of a rapid pandemic defense (Masoomikarimi 2021).

**Table 1:** Immunological therapeutic toolbox

Adequate diet and exercise
Screen and supplement vitamin deficiencies
Follow vaccination schemata
Heterologous vaccines intervention(s)
Convalescent plasma
Intravenous immunoglobulin
Monoclonal antibodies
Pro-inflammatory cytokines inhibitors (i.e., IL-6/ TNF inhibitors)
Complement inhibitors
Interferon therapy
Anti-inflammatory agents (i.e., Baricitinib)
Cellular therapy
Mesenchymal stem cells-based therapy
NK cell-based therapy
Unconventional therapies (i.e., fecal microbial transplant)

## Data Availability

Not application.

## Abbreviations

EIP: Extreme immunotherapies for pandemics
HVI: Heterologous vaccine interventions
IVIG: Intravenous immunoglobulin
mAbs: Monoclonal antibodies
Nabs: Neutralizing antibodies
NK: Natural killer
PLWH: Person living with HIV
WHO: World Health Organization

## References

[CR1] Bartoszko JJ (2021). Prophylaxis against covid-19: living systematic review and network meta-analysis. BMJ.

[CR2] Beigel JH (2020). Remdesivir for the treatment of COVID-19—final report. N Engl J Med.

[CR3] Boskovic M, Migo W, Likic R. SARS-CoV-2 mutations: a strain on efficacy of neutralizing monoclonal antibodies? Br J Clin Pharmacol. 2021. 10.1111/bcp.14849 PMC8251443 33847002

[CR4] Bruxvoort KJ (2019). Patient report of herpes zoster pain: Incremental benefits of zoster vaccine live. Vaccine.

[CR5] Brzoska J, von Eick H, Hundgen M (2020). Interferons in the therapy of severe coronavirus infections: a critical analysis and recollection of a forgotten therapeutic regimen with interferon beta. Drug Res (stuttg).

[CR6] Calabrese LH, Lenfant T, Calabrese C. Interferon therapy for COVID-19 and emerging infections: Prospects and concerns. Cleve Clin J Med. 2020. 10.3949/ccjm.87a.ccc066 33219050

[CR7] Davis MM, Tato CM, Furman D (2017). Systems immunology: just getting started. Nat Immunol.

[CR8] Dianzani F (1975). Viral interference and interferon. Ric Clin Lab.

[CR9] Dijksteel GS, Ulrich MMW, Middelkoop E, Boekema B (2021). Review: lessons learned from clinical trials using antimicrobial peptides (AMPs). Front Microbiol.

[CR10] Chambers ES et al. Vitamin D3 replacement enhances antigen-specific immunity in older adults. Immunother Adv 2021; 1. 10.1093/immadv/ltaa008 PMC9585673 36284901

[CR11] Fergie J, Srivastava A (2021). Immunity to SARS-CoV-2: lessons learned. Front Immunol.

[CR12] Group RC (2021). Dexamethasone in hospitalized patients with COVID-19. N Engl J Med.

[CR13] Guo H et al. Multi-omics analyses of radiation survivors identify radioprotective microbes and metabolites. Science. 2020; 370. 10.1126/science.aay9097 PMC7898465 33122357

[CR14] Haas EJ et al. Impact and effectiveness of mRNA BNT162b2 vaccine against SARS-CoV-2 infections and COVID-19 cases, hospitalisations, and deaths following a nationwide vaccination campaign in Israel: an observational study using national surveillance data. Lancet. 2021. 10.1016/S0140-6736(21)00947-8 PMC8099315 33964222

[CR15] Honjo K (2021). Convalescent plasma-mediated resolution of COVID-19 in a patient with humoral immunodeficiency. Cell Rep Med.

[CR16] Hupert N, Marin-Hernandez D, Nixon DF (2021). Can existing unrelated vaccines boost a COVID-19 vaccine prime?. EClinicalMedicine.

[CR17] James JS (1995). Animal cell transplantation: FDA meeting July 13 and 14. Food and drug administration. AIDS Treat News.

[CR18] Keller MD (2020). SARS-CoV-2-specific T cells are rapidly expanded for therapeutic use and target conserved regions of the membrane protein. Blood.

[CR19] Klassen SA (2021). The effect of convalescent plasma therapy on mortality among patients with COVID-19: systematic review and meta-analysis. Mayo Clin Proc.

[CR20] Kumar RN et al. Real-world experience of bamlanivimab for COVID-19: a case-control study. Clin Infect Dis. 2021 10.1093/cid/ciab305 PMC8083260 33846730

[CR21] La Manna MP (2020). Harnessing unconventional T cells for immunotherapy of tuberculosis. Front Immunol.

[CR22] Long BR (2008). Elevated frequency of gamma interferon-producing NK cells in healthy adults vaccinated against influenza virus. Clin Vaccine Immunol.

[CR23] Lucas C et al. Delayed production of neutralizing antibodies correlates with fatal COVID-19. Nat Med. 2021. 10.1038/s41591-021-01355-0 PMC8785364 33953384

[CR24] Marin-Hernandez D, Hupert N, Nixon DF (2021). The immunologists’ guide to pandemic preparedness. Trends Immunol.

[CR25] Market M (2020). Flattening the COVID-19 curve with natural killer cell based immunotherapies. Front Immunol.

[CR26] Masoomikarimi M (2021). Advances in immunotherapy for COVID-19: a comprehensive review. Int Immunopharmacol.

[CR27] McCann CD et al. A participant-derived xenograft model of HIV enables long-term evaluation of autologous immunotherapies. J Exp Med. 2021; 218. 10.1084/jem.20201908 PMC8129803 33988715

[CR28] Moorlag S, Arts RJW, van Crevel R, Netea MG (2019). Non-specific effects of BCG vaccine on viral infections. Clin Microbiol Infect.

[CR29] Nickbakhsh S et al. Virus-virus interactions impact the population dynamics of influenza and the common cold. Proc Natl Acad Sci U S A. 2019. 10.1073/pnas.1911083116 PMC6936719 31843887

[CR30] Opatowski L, Baguelin M, Eggo RM (2018). Influenza interaction with cocirculating pathogens and its impact on surveillance, pathogenesis, and epidemic profile: a key role for mathematical modelling. PLoS Pathog.

[CR31] Pearson H (2021). How COVID broke the evidence pipeline. Nature.

[CR32] Pistoia V (2018). Human γδ T-cells: from surface receptors to the therapy of high-risk leukemias. Front Immunol.

[CR33] Poccia F (2005). Vgamma9Vdelta2 T cell-mediated non-cytolytic antiviral mechanisms and their potential for cell-based therapy. Immunol Lett.

[CR34] Ragonnet-Cronin M (2021). Genetic evidence for the association between COVID-19 epidemic severity and timing of non-pharmaceutical interventions. Nat Commun.

[CR35] Ram DR, Manickam C, Lucar O, Shah SV, Reeves RK (2019). Adaptive NK cell responses in HIV/SIV infections: a roadmap to cell-based therapeutics?. J Leukoc Biol.

[CR36] Relf MV (1996). Xenotransplantation of baboon bone marrow cells: a historical review of the protocols as a possible treatment modality for HIV/AIDS. J Assoc Nurses AIDS Care.

[CR37] Rosenberg SA (2014). IL-2: the first effective immunotherapy for human cancer. J Immunol.

[CR38] Spinelli MA et al. Importance of non-pharmaceutical interventions in lowering the viral inoculum to reduce susceptibility to infection by SARS-CoV-2 and potentially disease severity. Lancet Infect Dis. 2021 10.1016/S1473-3099(20)30982-8 PMC7906703 33631099

[CR39] Starzl TE (1993). Baboon-to-human liver transplantation. Lancet.

[CR40] Wakao H, Sugimoto C, Kimura S, Wakao R (2017). Mucosal-associated invariant T cells in regenerative medicine. Front Immunol.

[CR41] Wang Z et al. MR1-restricted T cells: the new dawn of cancer immunotherapy. Biosci Rep 2020; 40. 10.1042/BSR20202962 PMC7670570 33185693

[CR42] WHO. Therapeutics and COVID-19: living guideline. 2021.

[CR43] WHO. Prioritizing diseases for research and development in emergency contexts. 2021.

[CR44] Wrangle JM (2018). ALT-803, an IL-15 superagonist, in combination with nivolumab in patients with metastatic non-small cell lung cancer: a non-randomised, open-label, phase 1b trial. Lancet Oncol.

[CR45] Xu D, Lu W (2020). Defensins: a double-edged sword in host immunity. Front Immunol.

[CR46] Zylberman V (2020). Development of a hyperimmune equine serum therapy for COVID-19 in Argentina. Medicina (b Aires).

